# The Effects of Omega-3 Fatty Acid Supplementation on Dexamethasone-Induced Muscle Atrophy

**DOI:** 10.1155/2014/961438

**Published:** 2014-05-25

**Authors:** Alan Fappi, Tiago S. Godoy, Jessica R. Maximino, Vanessa R. Rizzato, Juliana de C. Neves, Gerson Chadi, Edmar Zanoteli

**Affiliations:** Department of Neurology, University of São Paulo, No. 455, Dr. Arnaldo Avenue, 01246-903 São Paulo, SP, Brazil

## Abstract

Corticosteroids cause muscle atrophy by acting on proteasomal and lysosomal systems and by affecting pathways related to muscular trophysm, such as the IGF-1/PI-3k/Akt/mTOR. Omega-3 fatty acid (n-3) has been used beneficially to attenuate muscle atrophy linked to sepsis and cachexia; however, its effect on dexamethasone-induced muscle atrophy has not been evaluated. *Objectives*. We evaluated whether n-3 supplementation could mitigate the development of dexamethasone-induced muscle atrophy. *Methods*. Two groups of *Wistar* rats were orally supplemented with n-3 or vehicle solution for 40 days. In the last 10 days, dexamethasone, or saline solution, was administrated establishing four groups: control, dexamethasone, n-3, and dexamethasone + n-3. The cross-sectional areas of muscle fibers, gene expression (*MyoD, Myogenin, MuRF-1*, and *Atrogin-1*), and protein expression (Akt, GSK3*β*, FOXO3a, and mTOR) were assessed. *Results*. Dexamethasone induced a significant loss in body and muscle weight, atrophy in type 2B fibers, and decreased expression of P-Akt, P-GSK3*β*, and P-FOXO3a. N-3 supplementation did not attenuate the negative effects of dexamethasone on skeletal muscle; instead, it caused atrophy in type 1, 2A, reduced the expression of *Myogenin*, and increased the expression of *Atrogin-1*. *Conclusion*. Food supplements containing n-3 are usually healthful, but they may potentiate some of the side effects of glucocorticoids.

## 1. Introduction

Muscle atrophy is the loss of muscle mass resulting from a reduction in muscle fiber area or density due to a decrease in protein synthesis and an increase in muscle protein breakdown; these processes activate two major systems: the proteasomal (ubiquitin-proteasome system or UPS) and the autophagic-lysosomal system [1–3]. Many conditions are associated with skeletal muscle atrophy, such as prolonged fasting, inactivity, or pathological conditions such as sepsis, cachexia, AIDS, cancer, and glucocorticoid treatment among others [1, 2].

Glucocorticoids are one of the most widely prescribed therapeutic compounds, used in the treatment of inflammatory, autoimmune, lymphoproliferative, and neuromuscular disorders [4, 5]. Despite its benefits in inflammatory responses, treatment with glucocorticoids harms the skeletal muscle [6].

Many studies have shown that glucocorticoids not only cause muscle atrophy through the IGF-1/PI-3K/Akt/mTOR and myostatin/Smad2/3 pathways [7, 8], but also through the regulation of muscle transcription factors, atrogenes, and cathepsins [8, 9]. Some of these mechanisms involve the reduction in mTOR activity induced by an increase of REDD1 (regulated in development and DNA damage responses 1) [10], and an increase in the GSK3*β* protein. Increased levels of GSK3*β* are associated with the proteasomal system leading to increased protein degradation and decreased protein synthesis [11]. Glucocorticoids reduce the activity of Akt, which, among other functions, acts as a negative regulator of the forkhead transcription factor (FOXO). FOXOs activation leads to a rapid protein degradation and stimulates the transcription of several components of the lysosomal and ubiquitin-proteasome systems [12–16]. Activated FOXO1 and FOXO3a lead to the upregulation of* Atrogin-1*,* MuRF-1*,* REDD1*, and* 4EBP1*, which lead to muscle atrophy [17].

Polyunsaturated fatty acids (PUFAs) are considered beneficial food supplements to human health, particularly effective in the cardiovascular and central nervous systems [18–20]. Two particular PUFAs families the Omega-3 (n-3) and Omega-6 (n-6) fatty acids are the most consumed worldwide and are linked to effects widely discussed in the scientific community. The n-3 as supplement is commercialized worldwide with free access of the population in capsules ranging from 500–2000 mg of fish oil extract per capsule. The World Health Organization (WHO) considers 0.25 to 2 g of EPA/DHA per day as part of a healthy diet [21], and the US Food and Drug Administration (FDA) recommends a maximum intake of 3 g per day of n-3 (EPA/DHA) [22], equivalent to 50 mg/kg/day in a 60 kg person. The American Heart Association (AHA) recommends the intake of 2 to 4 g of EPA/DHA per day to patients with increased levels of serum triglycerides, provided as capsules under a physician's care [23]. The n-3 PUFAs such as EPA (eicosapentaenoic acid), DHA (docosahexaenoic acid), and ALA (*α*-linolenic acid) are incorporated into the cell membrane after absorption and can modulate various cellular functions, such as signaling and gene expression [24] and decrease the activity of certain UPS components as seen in cancer cachexia [25]. However, to our knowledge, the effects of n-3 PUFA on glucocorticoid-induced muscle atrophy have not been tested.

This study evaluated the effects of n-3 PUFA (capsule formulation) on the development of dexamethasone-induced muscle atrophy through histological analysis, expression of genes involved in protein synthesis and degradation, and assessment of expression of proteins involved in the IGF-1/PI-3k/Akt/mTOR pathway.

## 2. Methods

### 2.1. Animal Treatment

We used in this study 24 male* Wistar* rats, aged between 10 to 12 weeks, with body weights from 330 to 370 grams. All experiments were performed according to the National Institutes of Health (NIH) guidelines on care, handling, and use of laboratory animals and approved by the local ethics committee. Animals were housed in four cages (6 animals each) and placed under controlled light (12-hour light/dark cycle) and temperature (25°C) conditions. Animals were fed* ad libitum* with a standard commercial diet (Nuvilab CR1) with an approximated composition of 22.0% crude protein, 4.5% ether extract, 8.0% fibrous matter, 1.4% calcium, and 0.8% phosphorus and amino acids (DL-Methionine 300 mg, lysine 100 mg).

In the first part of the study, 24 animals were divided into two initial groups: 12 animals were supplemented daily for 40 days, via gavage (v.g.), with n-3 capsules (Proepa, Ache laboratories) diluted in vehicle solution (tween-20 and ultrapure water) and the other 12 animals received the same volume of vehicle solution for the same period of time. According to the manufacturer, each n-3 capsule contained energetic value (9.4 Kcal = 39.5 KJ); total fats (1.0 g); monounsaturated fats (0.3 g); polyunsaturated fats (0.4 g); eicosapentaenoic acid (180 mg), and docosahexaenoic acid (120 mg). The amount of EPA and DHA administrated was adjusted in vehicle solution to the dosage of 100 mg/kg/day per animal. After day 30, six animals from each group received 5 mg/kg/day of subcutaneous (sc.) dexamethasone (Decadron, Ache) for 10 days to induce muscle atrophy, along with n-3 supplementation. The six remaining animals in each group received saline solution as a control. The experimental protocols utilized in these four groups are summarized as DX + n-3 group [n-3 PUFA v.g. (40 days) + dexamethasone sc. (last 10 days)], n-3 group [n-3 PUFA v.g. (40 days) + saline solution sc. (last 10 days)], DX group [vehicle solution v.g. (40 days) + dexamethasone sc. (last 10 days)], and CT group [vehicle solution v.g. (40 days) + saline solution sc. (last 10 days)]. The animals were weighed on days 1, 10, 20, 30, 32, 34, 36, and 40.

Animals were euthanized after 40 days by intraperitoneal injection with sodium pentobarbital (30 mg/kg). Bilateral gastrocnemius (GA) and tibialis anterior (TA) muscles were collected and weighed. Right-side samples were mounted in Tissue-Tek, immersed in isopentane, and cooled in liquid nitrogen for histological analysis. The remaining muscles were frozen in liquid nitrogen and stored at −80°C for subsequent gas chromatography and extraction of protein and RNA.

### 2.2. Analysis of PUFAs and Fat Content by Gas Chromatography

GA muscle samples were analyzed by gas chromatography to evaluate PUFAs and fat content. Fat extraction was carried out by the Bligh and Dyer methodology [26] according to Iverson et al. (2001) [27]. Initially, 125 mg of samples was homogenized (Ultra-turrax, IKA T10 Basic) with a mixture containing chloroform (125 *μ*L) and methanol (200 *μ*L). The solution was homogenized again with chloroform (125 *μ*L) and saline (125 *μ*L of 0.88% NaCl). The final emulsion was centrifuged at 13,000 g for 5 minutes at 25°C. The upper phase was removed and the lower phase evaporated under nitrogen stream. Fat samples were derivatized using the technique of direct esterification described by Shirai et al. (2005) [28] and their composition was determined by gas chromatograph (Agilent 7890 A GC System, Agilent Technologies, Inc., Santa Clara, USA). A fused silica capillary column (J&W DB-23 Agilent 122-236; 60 m × 250 mm inner diameter) was used for injection. High-purity helium was used as the carrier gas at a flow rate of 1 mL/minute and a split injection of 50 : 1. The programming of column temperature was starting at 80°C; a gradient step at a heating rate of 5°C/minute to reach 175°C; a gradient step at a heating rate of 3°C/minute to reach 230°C; and maintenance of 230°C for 5 minutes. The injector and detector (FID) temperatures were 250°C and 280°C, respectively. The fatty acids were identified by comparing their retention times to those of four purified standard mixtures of fatty-acid methyl esters (Sigma Chemical Co.: 4-7801, 47085-U, 49453-U, and 47885-U). The results were expressed as percentages of total fatty acids. Muscle tissue contents of total PUFAs and fat were determined. DX group muscles were not evaluated because this group did not receive n-3 supplementation.

### 2.3. Analysis of Cross-Sectional Areas in the Skeletal Muscle

Cross-sectional areas from muscle fibers (types 1, 2A, and 2B) were calculated and analyzed. TA muscles were sectioned in a Leica CM3000 cryostat (8 *μ*m) and sections were treated using the metachromatic dye-ATPase method (mATPase) according to Ogilvie and Feeback (1990) [29]. Slides with dried frozen sections were placed in a preincubation solution composed of distilled water (475 mL), dehydrated potassium acetate (2.45 g), and calcium chloride (1.30 g) at pH 4.5 for 2 minutes; slides were subsequently rinsed in Tris buffer. Slides were then incubated for 25 minutes in an incubation solution composed of distilled water (30 mL), potassium chloride (185 mg), ATP, disodium salt (76 mg), dehydrated calcium chloride, (0.18 M = 5 mL), and Sigma 221 buffer (1.5 M 2-amino-2-methyll-propanol) (3.35 mL) at pH 9.4. Slides were rinsed by dipping in three changes of 1% calcium chloride solution, stained with 1% aqueous toluidine blue for 10 seconds, and immediately rinsed in distilled water. Images of stained muscle sections were acquired on an Olympus AX70 light microscope (Olympus Melville, NY). Cross-sectional areas from types 1, 2A, and 2B fibers, from five slides per animal (300 to 400 fibers/animal), were measured using the Image J software (NIH).

### 2.4. Immunoblot Expression of mTOR, Akt, GSK3*β*, and FOXO3a Proteins

The mTOR, Akt, GSK3*β*, and FOXO3a protein levels were assessed by quantitative Western blot analysis in GA muscle tissue samples collected as described. Tissues were homogenized in a Potter homogenizer in lysing buffer containing 1% protease and phosphatase inhibitor cocktail (Sigma-Aldrich; P5726 and A-1153, resp.), 1 mM EDTA (Sigma), and 1% NP40 (Sigma) diluted in phosphate-buffered saline (PBS, pH 7.4) and stored at −80°C until use. Total protein concentrations were quantified by Bradford method (1976) [30]. Samples with up to 50 to 100 *μ*g of total protein were submitted to Western blot. The primary antibodies used were Akt (1 : 2,000, Cell Signaling #4691), P-Akt (1 : 2,000, Cell Signaling #4060, Ser473); mTOR (1 : 500, Cell Signaling #2972), P-mTOR (1 : 500, Cell Signaling #2971, Ser2448), GSK3*β* (1 : 2,000, Cell Signaling #9315), P-GSK3*β* (1 : 2,000, Cell Signaling #9322, Ser9), FoXO3a (1 : 500, Cell Signaling #2497), P-FoXO3a (1 : 500, Cell Signaling #9466, Ser253), and *α*-tubulin (1 : 30,000, Hybridoma bank) in 5% BSA/TBS-T. The secondary conjugated antibodies were IgG-ECL anti-rabbit (1 : 10,000, KPL) or anti-mouse (1 : 6,000, Santa Cruz Biotechnology). Finally, membranes were incubated with Western Lightning Chemiluminescence Reagent Plus (PerkinElmer Life Science, USA) for 1 min and exposed to an X-ray film (Hyperfilm ECL, Amersham Biosciences, USA) for visualization of the protein bands. The films were scanned (HP Scanjet G4000 series) and protein levels were quantified by densitometry using a computer-assisted image analyzer through the Image J software (version 1.43u, National Institute of Health, USA). The density values were normalized by the *α*-tubulin density values.

### 2.5. Expression of* MyoD*,* Myogenin*,* REDD1*,* REDD2*,* MuRF-1*, and* Atrogin-1* Measured by Quantitative PCR

Total RNA was extracted from GA muscle samples with TRIZOL reagent (Invitrogen), according to the manufacturer's instructions. RNA quantity and integrity were assessed by spectrophotometry (Nanodrop, Thermo Scientific, USA) and microfluidics-based electrophoresis (Agilent 2100 Bioanalyzer, Agilent Technologies, USA), respectively. RNA samples with 260/280 OD ratios of approximately 2.0 and RIN > 7.0 were used in the quantitative PCR (qPCR) reactions. cDNA was synthesized from 1 *μ*g of total RNA using TaqMan Reverse Transcription Reagents N808-0234 (Applied Biosystems Life Technologies), according to the manufacturer's instructions.

The qPCR reactions were carried out in duplicate with 50 ng cDNA, SYBR Green PCR master mix (Fermentas), and 800 to 900 nM of each primer in a final volume reaction of 25 *μ*L in the* Step One Plus* device (Applied Biosystems, USA). Real-time PCR was performed to investigate the expression of the following genes:* MyoD* [31] (*forward*: 5′-TGTAACAACCATACCCCACTCTC-3′*, reverse*: 5′-AGATTTTGTTGCACTACACAGCA-3′),* Myogenin* [32] (*forward*: 5′-CACATCTGTTCGACTCTCTTCT-3′;* reverse*: 5′-ACCTTGGTCAGATGACAGCTTTA-3′),* REDD-1* [33] (*forward*: 5′-CACCGGCTTCAGAGTCATCA-3′;* reverse*: 5′-CGGGTCTCCACCACA GAAAT-3′),*REDD-2* [33] (*forward*: 5′-CTTCAGCGTCTGGTGAAATCC-3′;* reverse*:* 5*′-ATGCTGGCCGTGTTCTTACTG-3′),* MuRF-1* [34] (*forward*: 5′-TCGACATCTACAAGCAGGAA-3′;* reverse*: 5′-CTGTCCTTGGAAGATGCTTT-3′), and* Atrogin-1* [34] (*forward*: 5′-TGAAGACCGGCTACTGTGGAAGAGAC-3′;* reverse*: 5′-TTGGGGTGAAAGTGAGACGGAG CAG-3′). The results were normalized individually by the expression of GAPDH [35] (*forward*: 5′-ACGCCAGTAGACTCCACGAC-3′;* reverse*: 5′-ATGACTCTACCCACGGCAAG-3′) and further compared by logarithmic normalization (2^−ΔΔCT^, ABI PRISM 7700 Sequence Detection System protocol; Applied Biosystems).

### 2.6. Statistical Analysis

Differences between means were analyzed by unpaired Student's* t*-test, whereas differences among means were analyzed by One- or Two-way ANOVA followed by Bonferroni post hoc testing. All analyses were performed using the Graph Pad Prism 5.0 software for Windows (Graph Pad Software, USA). The data are presented as mean ± S.E.M., with the significance level set at *P* < 0.05.

## 3. Results

### 3.1. n-3 PUFA Does Not Alleviate Body and Muscle Weight Loss or Muscle Atrophy Induced by Dexamethasone

**Figure 1:**
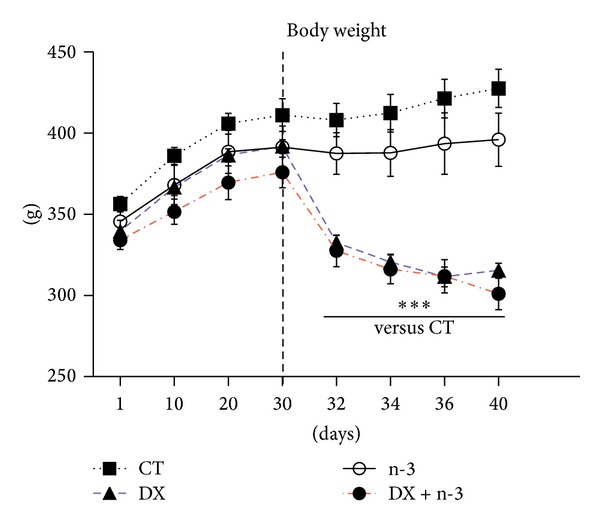
Changes in body weight (grams) in animals treated with n-3 PUFA or vehicle for 40 days and dexamethasone or vehicle (last 10 days). The dotted line indicates the start of dexamethasone administration. CT = control group (*n* = 6), DX = dexamethasone group (*n* = 5), n-3 = n-3 PUFA group (*n* = 6), and DX + n-3 = dexamethasone + n-3 PUFA group (*n* = 6). Values represent means ± S.E.M., analyzed by two-way ANOVA repeated measure followed by Bonferroni post hoc test, ***P < 0.001.

All animals presented appropriate weight gain according to natural growth during the first 30 days of the study with no statistical difference in weight gain between the four experimental groups during this period. A significant difference in body weight became evident on the second day after dexamethasone administration (groups DX and DX + n-3) (Figure 1). One animal from the DX group died on the fifth day of dexamethasone administration. The mean body weight in the CT group was 356.33 ± 4.7 g at the beginning of the study and 427.66 ± 11.8 g at the end of the study, indicating a gain of 20.01%. The weight gain in the n-3 group was 14.61% (from 345.5 ± 8.8 g to 396.0 ± 16.4 g), which has no statistical difference to CT group. Animals from the DX and DX + n-3 groups showed both body weight loss compared to the initial to the final day of study, corresponding 8.54% in the DX group (initial weight 339.5 ± 6.8 g and final weight 310.5 ± 5.6 g) and 9.88% in the DX + n-3 group (initial weight 334.00 ± 5.52 g and final weight 301.0 ± 9.8 g). Whereas the body weight loss pre- and postdexamethasone administration was of 21.75% (DX group) and 19.91% (DX + n-3 group), both significantly decreased compared to CT group (*P* < 0.001). There were no statistical differences in body weight comparing DX to the DX + n-3 group and comparing CT to the n-3 group.

**Figure 2:**
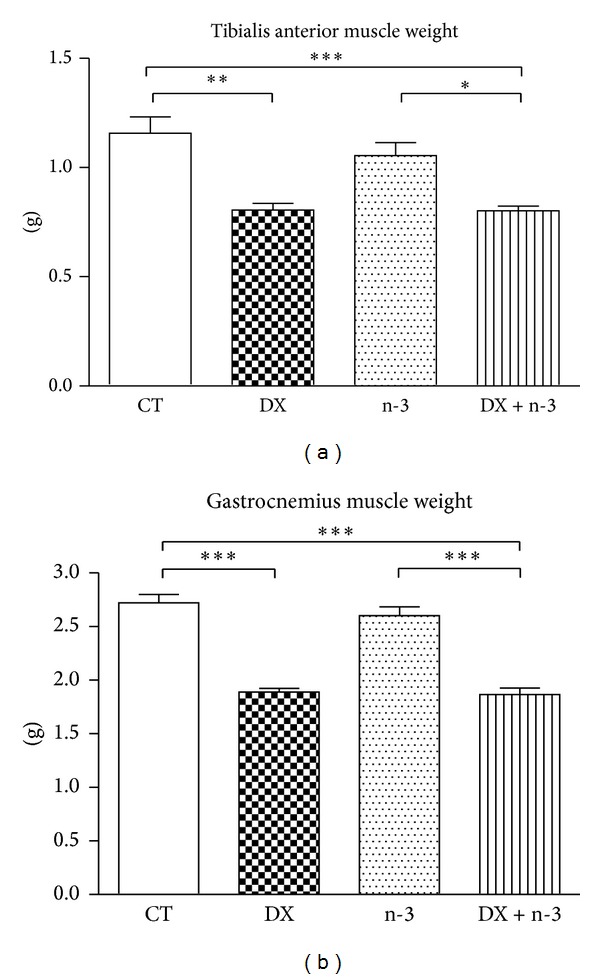
Muscle weights in the DX and DX + n-3 groups were significantly decreased compared to the CT group. CT = control group (*n* = 6), DX = dexamethasone group (*n* = 5), n-3 = n-3 PUFA group (*n* = 6), and DX + n-3 = dexamethasone + n-3 PUFA group (*n* = 6). Values represent means ± S.E.M., analyzed by one-way ANOVA followed by Bonferroni post hoc test, *P < 0.05; **P < 0.01, and ***P < 0.001.

The TA and GA muscle samples were weighed immediately after euthanasia; the weights of muscle samples from the DX and DX + n-3 groups was significantly smaller than those from the CT group (*P* < 0.01 and *P* < 0.001, resp.) (Figure 2). There was no difference in muscle weight comparing CT to the n-3 group. These findings indicate that the administration of n-3 did not effectively mitigate the weight loss either in body or muscle tissues induced by dexamethasone.

**Figure 3:**
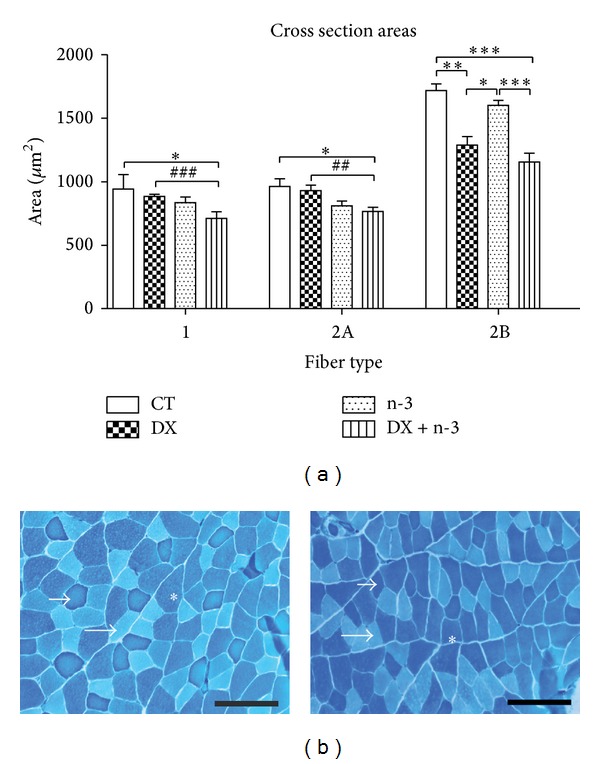
Means of cross-sectional areas of tibialis anterior muscle fibers (type 1, 2A, and 2B) in rats treated with n-3 PUFA or vehicle for 40 days and dexamethasone or vehicle (last 10 days). (a) Fiber type size distribution; (b) representative mATPase dye histological images of TA muscle from the control group (left), and from the DX + n-3 group (right). Small arrow = type 1 fiber; large arrow = type 2A fiber; asterisk = type 2B fiber. Scale bar (100 *μ*m). CT = control group (*n* = 6), DX = dexamethasone group (*n* = 5), n-3 = n-3 PUFA group (*n* = 6), and DX + n-3 = dexamethasone + n-3 PUFA group (*n* = 6). Values represent means ± S.E.M., analyzed by One-way ANOVA (^∗^) followed by Bonferroni post hoc test or unpaired Student's* t*-test (^#^), *P < 0.05; ^∗∗^ or ^##^ *P* < 0.01, and ^∗∗∗^ or ^###^ *P* < 0.001.

In the muscle atrophy analysis, we observed that the administration of dexamethasone induced a significant TA muscle atrophy of type 2B muscle fibers (fast-twitch), reducing their cross-sectional area by 25.03% in the DX group and by 32.71% in the DX + n-3 group compared to the CT group (*P* < 0.01 and *P* < 0.001, resp.). In addition, the DX + n-3 group showed a significant muscle atrophy of type 1 and 2A muscle fibers compared to the CT (24.66% and 20.53%, resp.) (*P* < 0.05) and to the DX group (19.62% and 17.95% in fibers 1 and 2A, resp.) (*P* < 0.001 and *P* < 0.01, resp.), (Figures 3(a) and 3(b)). These findings confirmed that the administration of dexamethasone induces atrophy, preferentially in type 2B muscle fibers. In addition, the concomitant administration of n-3 PUFA induced atrophy in muscle fiber types that are usually more resistant to atrophy induced by dexamethasone, such as type 1 and 2A fibers.

### 3.2. n-3 PUFA Induces an Increase in PUFA and ALA and a Decrease in Fat and AA in Skeletal Muscles

**Figure 4:**
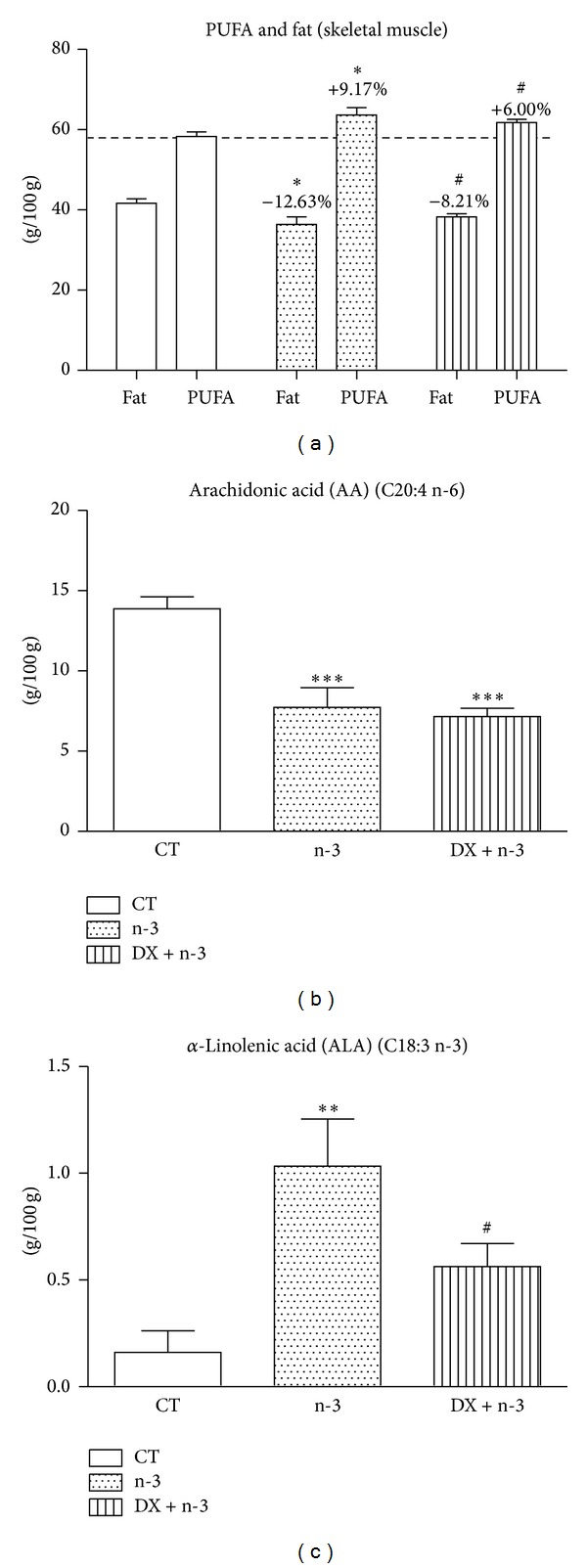
Gas chromatography of GA muscle samples from rats treated with n-3 PUFA or vehicle for 40 days and dexamethasone or vehicle (last 10 days). (a) Percentages of total fat and PUFAs per experimental group. Percentages above the bar indicate decrease or increase in PUFA and fat content compared to the same type of fatty acid in the CT group. ((b) and (c)) percentages of arachidonic and *α*-linolenic acids, respectively. CT = control group (*n* = 6), n-3 = n-3 PUFA group (*n* = 6), and DX + n-3 = dexamethasone + n-3 PUFA group (*n* = 6). Statistical analysis is in comparison with the CT group. Values represent means ± S.E.M., analyzed by one-way ANOVA (^∗^) followed by Bonferroni post hoc test or Student's* t*-test (^#^), ^∗^ or ^#^ *P* < 0.05; **P < 0.01 and ***P < 0.001.

PUFAs content increased by 9.17% and fat content decreased by 12.63% in the n-3 group, showing a significant elevation compared to the CT group (*P* < 0.05). Similar increase in PUFAs and decrease in fat content was observed in the DX + n-3 group compared to the CT group, (+6.00% and −8.21%, resp.) (*P* < 0.05) (Figure 4(a)).

We observed a significant decrease in the arachidonic acid (AA) content in the n-3 and DX + n-3 groups compared to the CT group (*P* < 0.001) (Figure 4(b)), and the *α*-linolenic acid (ALA) content was significantly increased in the n-3 group compared to the CT group (*P* < 0.01) with a mild increase in the DX + n-3 group compared to the CT group (*P* < 0.05) (Figure 4(c)). These findings indicated that the oral administration of n-3 PUFA was effective for the incorporation of PUFAs in the skeletal muscle.

### 3.3. The Concomitant Administration of Dexamethasone and n-3 PUFA Induces Suppression of Muscle Transcriptional Factors

**Figure 5:**
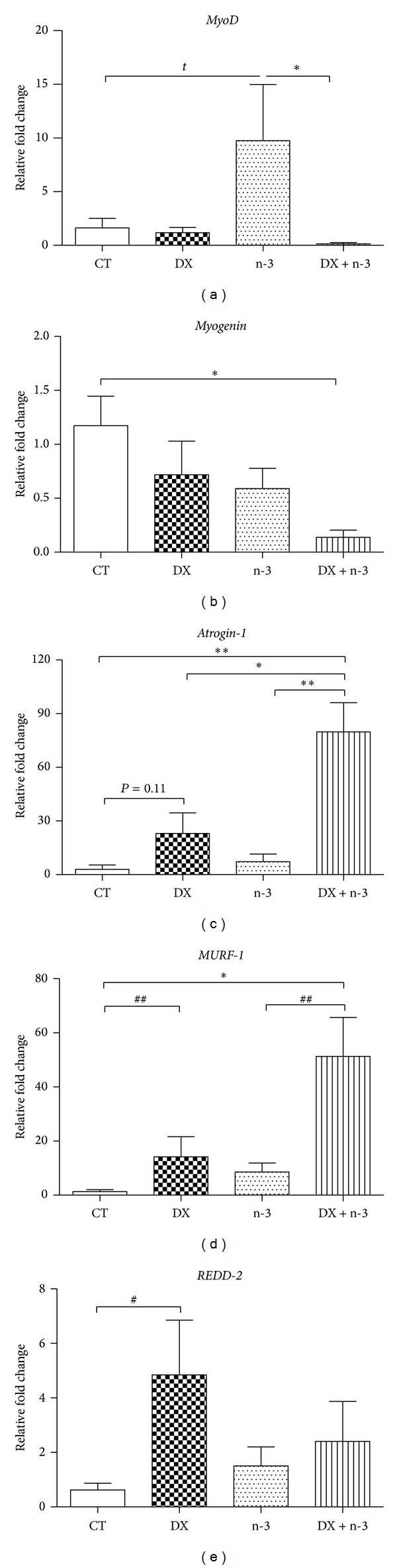
Quantitative PCR results of gene expression in GA muscle samples from rats treated with n-3 PUFA or vehicle for 40 days and dexamethasone or vehicle (last 10 days). Relative fold change values for (a)* MyoD*, (b)* Myogenin,* (c)* Atrogin-1*, (d)* MuRF-1*, and (e)* REDD2.* Results are represented as means ± S.E.M. CT = control group (*n* = 6), DX = dexamethasone group (*n* = 5), n-3 = n-3 PUFA group (*n* = 6), and DX + n-3 = dexamethasone + n-3 PUFA group (*n* = 6). Values represent means ± S.E.M., analyzed by one-way ANOVA (^∗^) followed by Bonferroni post hoc test or Student's* t*-test (^#^), ^∗^ or ^#^ *P* < 0.05 and ^##^ *P* < 0.01.

The effects of dexamethasone and n-3 PUFA administration on muscle regeneration were evaluated through the expression of muscle transcriptional factors. The expression of* MyoD* was increased significantly in the n-3 group only when compared to the DX + n-3 group (*P* < 0.05) (Figure 5(a)), suggesting that dexamethasone abrogated the expression of* MyoD* through interaction with n-3. Conversely,* Myogenin* expression was significantly decreased in the DX + n-3 group in comparison to the CT group (*P* < 0.05) (Figure 5(b)). In DX group, there were no statistical differences in the expressions of* MyoD* or* Myogenin* as observed in DX + n-3 group. These results suggest that the administration of n-3 PUFA together with dexamethasone may affect the muscular regeneration process.

### 3.4. Dexamethasone Associated with n-3 PUFA Induces Higher* Atrogin-1* Expression in Muscle Fibers Than Dexamethasone Alone

We evaluated the expression of the* Atrogin-1* and* MuRF-1* atrogenes after dexamethasone and n-3 administration. Both genes showed increased expression in the DX + n-3 group compared to the CT group (*P* < 0.01 and *P* < 0.05, resp.), as well as in the DX group in* MuRF-1* expression (*P* < 0.01). The n-3 group did not show any increase in atrogenes expression (Figures 5(c) and 5(d)). The expression of* MuRF-1* in the DX + n-3 group was 2.74-fold higher than DX group (not statistically different), and in* Atrogin-1* expression was 3.46-fold higher (*P* < 0.05). The expression of* REDD2* genes was increased significantly only in the DX group compared to the CT group (*P* < 0.05) without any increase in DX + n-3 group (Figure 5(e)). No significant difference in* REDD1* expression was observed between the groups (data not shown). These data suggest that dexamethasone administration may affect REDD2 and atrogenes expression in skeletal muscles and that the concomitant administration of n-3 PUFA can increase this effect in* Atrogin-1* expression.

### 3.5. Dexamethasone Induces a Decrease in Akt, GSK3-*β*, and FOXO3a Phosphorylation

**Figure 6:**
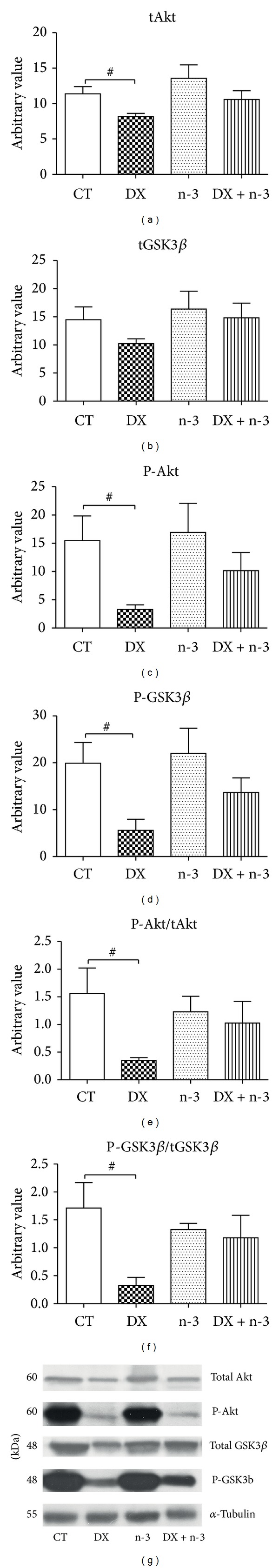
Protein expression in GA muscle samples from rats treated with n-3 PUFA or vehicle for 40 days and dexamethasone or vehicle (last 10 days). Relative optical density of total Akt (tAkt) and phosphorylated Akt (P-Akt) ((a) and (c)), total GSK3*β* (tGSK3*β*) and phosphorylated GSK3*β* (P-GSK3*β*) ((b) and (d)), and tAkt/P-Akt and tGSK3*β*/P-GSK3*β* ratios ((e) and (f), resp.). Bands representing tAkt, P-Akt, tGSK3, and P-GSK3*β* are illustrated in (g). *α*-Tubulin (55 kDa) was used as control. The numbers represent the mean ± S.E.M. CT = control group (*n* = 6), DX = dexamethasone group (*n* = 5), n-3 = n-3 PUFA group (*n* = 6), and DX + n-3 = dexamethasone + n-3 PUFA group (*n* = 6). Values represent means ± S.E.M., analyzed by Student's* t*-test, ^#^ *P* < 0.05.

**Figure 7:**
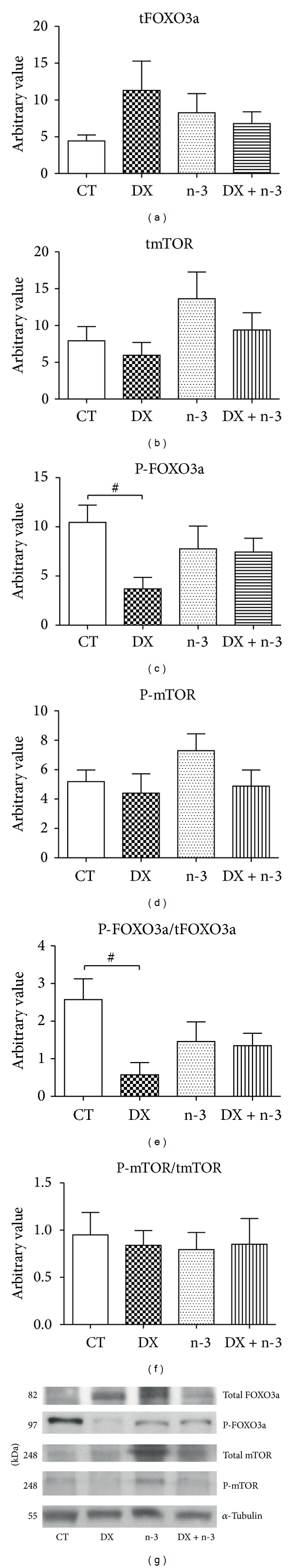
Protein expression in GA muscle samples from rats treated with n-3 PUFA or vehicle for 40 days and dexamethasone or vehicle (last 10 days). Relative optical density of total FOXO3a (tFOXO3a) and phosphorylated FOXO3a (P-FOXO3a) ((a) and (c)), total mTOR (t-mTOR) and phosphorylated (P-mTOR) ((b) and (d)), and P-FOXO3a/tFOXO3a and P-mTOR/tmTOR ratios ((e) and (f), resp.). Bands representing tFOXO3a, P-FOXO3a, t-mTOR, and P-mTOR are illustrated in (g). *α*-Tubulin (55 kDa) was used as control. The numbers represent the mean ± S.E.M. CT = control group (*n* = 6), DX = dexamethasone group (*n* = 5), n-3 = n-3 PUFA group (*n* = 6), and DX + n-3 = dexamethasone + n-3 PUFA group (*n* = 6). Values represent means ± S.E.M., analyzed by Student's* t*-test, ^#^ *P* < 0.05.

We evaluated the phosphorylated (P), total (t), and phosphorylated/total ratio forms of Akt, GSK3*β*, FOXO3a, and mTOR proteins in GA muscle samples to assess protein expression in the IGF-1/PI-3K/Akt/mTOR pathway (Figures 6 and 7).

In the DX group, the t-AKT, P-Akt, and P-GSK3*β* expression, as well as, P-Akt/tAkt and P-GSK3*β*/tGSK3*β* ratios were significantly decreased compared to the CT group (*P* < 0.05) (Figures 6(a), 6(c), 6(d), 6(e), and 6(f)).

The P-FOXO3a and P-FOXO3a/tFOXO3a ratio were significantly decreased (*P* < 0.05) compared to the CT group (Figures 7(c), and 7(e)). No significant difference was observed in mTOR expression between the four groups (Figures 7(b), 7(d), and 7(f)).

DX + n-3 group showed intermediate values in the expression of P-Akt and P-FOXO3a between CT and DX groups, however, with no statistical difference comparing both groups. The expression of all forms of Akt, GSK3*β*, mTOR, and FOXO3a was not affected in the groups supplemented with n-3. These findings suggest that the phosphorylation of Akt, GSK3*β*, and FOXO3a is inhibited by dexamethasone administration, and that the n-3 supplementation, apparently, contributes to attenuate these protein changes.

## 4. Discussion 

Some studies have shown that EPA and n-3 PUFA supplementation can alleviate muscle atrophy related to cancer, fasting, and septicemia [36–38]. Therefore, we sought to verify whether n-3 would influence the development of muscle atrophy induced by dexamethasone. We observed that dexamethasone administration induced significant loss of body and muscle weight and caused important muscle atrophy in type 2B fibers in rats and that the concomitant supplementation of n-3 did not attenuate these alterations.

Other studies have shown that n-3 is absorbed effectively by skeletal muscles when administrated orally [39–41]. In our study, the gas chromatography analysis showed that n-3 supplementation for 40 days was effective to increase the levels of PUFAs and fatty acids in the skeletal muscle. Thus, we believe that the lack of an effective n-3 attenuation of the dexamethasone-induced muscle atrophy was not related to deficient intestinal absorption of n-3. The dose of n-3 used in our study, similar to that recommended by the US FDA, WHO, and AHA for humans (approximated ratio of 50 mg/kg/day) [21–23], could not have been effective; however, much higher dose of n-3 (EPA), up to 2.5 g/kg/day, has been tried by others [42]. Thus, further studies testing different concentrations of n-3 and dexamethasone are necessary to confirm the effects of n-3 in the skeletal muscle under dexamethasone treatment found in the present study.

The 2B muscle fiber atrophy observed in this study has already been described as resulting from dexamethasone administration [43]. Some studies postulate that the high sensitivity of 2B fibers is due to the lower content of PGC1*α* in this type of fiber [44]. Interestingly, in our study, the concomitant administration of n-3 induced significant muscle atrophy of types 1 and 2A muscle fibers, which are usually more resistant to dexamethasone.

According to another study, AA (arachidonic acid) levels stimulate an increase of protein synthesis with release of PGF2*α* [45]. Sohal et al., 1992 [46], showed that EPA administration, by changing fatty acid composition of muscle phospholipids, causes a decrease in PGF2*α* synthesis, and this change might suppress the rate of protein synthesis on the muscles. AA is the immediate precursor of PGF [47], and EPA/DHA administration is associated with a decrease in levels of AA [48]. In our study we observed a reduction of 42% in AA levels in the animals treated with n-3 and dexamethasone. This finding might indicate a possible double effect on the muscles of these animals, with a reduction of protein synthesis (by n-3 supplementation, which reduces AA levels) and increase of protein degradation (by dexamethasone administration, which inhibits IGF-1 pathway-related proteins).

Concerning cell signaling mechanisms, Le Foll et al. (2007) [40] showed that the administration of a diet rich in n-3 to rats eliminates insulin's ability to stimulate PI-3K activity and slightly reduces the level of insulin-induced Akt phosphorylation in muscle tissue. The decrease in Akt activity by PI-3k results in a higher activation of transcription factors, such as FOXOs, which might lead to a higher transcription of E3 ligases involved in the muscle atrophy process [14]. According to the literature, the atrophy induced by glucocorticoids occurs through genetic and molecular mechanisms involved in protein degradation, including reduction in Akt, GSK3*β*, P70S6K, and mTOR phosphorylation and increase in the transcription of atrogenes [49, 50]. In our study, the levels of Akt, GSK3*β*, and FOXO3a phosphorylation were decreased in the DX group; in addition, DX + n-3 group showed higher activation of atrogenes such as* Atrogin-1 and MuRF-1*. This last finding indicates that the supplementation of n-3 might aggravate the side effects of dexamethasone by affecting upstream pathways of atrogenes activation. Considering that many studies have reported that the administration of n-3 alters the lipid composition in cell membranes [46, 51], we might speculate that the n-3 supplementation influences dexamethasone-induced muscle atrophy, affecting lipid composition in the sarcolemma and leading to decreased protein synthesis associated with the effects of hypercortisolism on skeletal muscle. These alterations could have modified the activity of receptors in cell membranes, thus altering the activation of intracellular pathways and downstream regulatory proteins, such as those related to the IGF-1/PI3-K/Akt/mTOR pathway, which are compartmentalized and activated through their translocation to the plasma membrane [52]. Nevertheless, these effects are tissue- and cell-specific, because activation or inactivation of Akt by the administration of fatty acids seems to be cell-type dependent [40].

Wang et al., (2006) [10], showed that dexamethasone increases* REDD1* expression in skeletal muscle, which subsequently activates the tuberin-hamartin complex and suppresses mTOR activity. Moreover, the same study observed a decrease in* REDD2* mRNA expression. Controversially, Frost et al. (2009) [53] showed that IGF-1, a stimulator of muscle synthesis, increases* REDD1* gene and protein expression in skeletal muscle with a paradoxically greater protein synthesis in myotubes expressing more* REDD1*. The expression of* REDD2*, in this same study was decreased by IGF-1 administration. In our study,* REDD1* expression did not show consistent changes; however,* REDD2* expression increased significantly in the DX group compared to the control group.

The reduction in* Myogenin* levels, observed in the DX + n-3 group, is consistent with results from other studies showing that dexamethasone induces many unfavorable conditions to myogenesis, partially through* Myogenin* inhibition [54, 55], but we did not observe a similar increase in DX group. Another study showed that dexamethasone prevents the formation of TNF-alpha and the release of lipopolysaccharide-stimulated IL-6, which influences myoblast proliferation [56]. However, no change was demonstrated in transcription factors related to myogenesis in DX group; a decrease was observed only in DX + n-3 group compared to the CT group. The increased* MyoD* expression observed in the n-3 group is consistent with results reported by Castillero et al. (2009) [57], which showed an increased* MyoD* expression in normal and arthritic rats after EPA administration, without changes in* Myogenin* levels.

In conclusion, this study confirms the deleterious effects of corticosteroids on skeletal muscle. As this type of medicine is commonly used to treat several medical conditions, the identification of drugs or nutritional supplements that could overcome these deleterious effects would be extremely useful in clinical practice. Furthermore, this study highlights that n-3 fatty acids (EPA/DHA) supplementation in rats did not attenuate the negative effects of dexamethasone on skeletal muscle, showing in addition atrophy on type 1 and 2A muscle fibers, increased atrogene expression, and reduced* Myogenin* levels. Thus, food supplements such as n-3, usually considered healthful, may potentiate some of the side effects of glucocorticoids.
